# Supplementary material for: Crystallization Induced Enhanced Emission in Two New Zn(II) and Cd(II) Supramolecular Coordination Complexes with the 1-(3,4-Dimethylphenyl)-5-Methyl-1H-1,2,3-Triazole-4-Carboxylate Ligand
Source: Polymers (Basel). 2020 Aug 6;12(8):1756. doi: 10.3390/polym12081756 (PMC7464244; doi:10.3390/polym12081756)
Supplement: Supplementary file 1 [file polymers-12-01756-s001.pdf]

Supporting information.

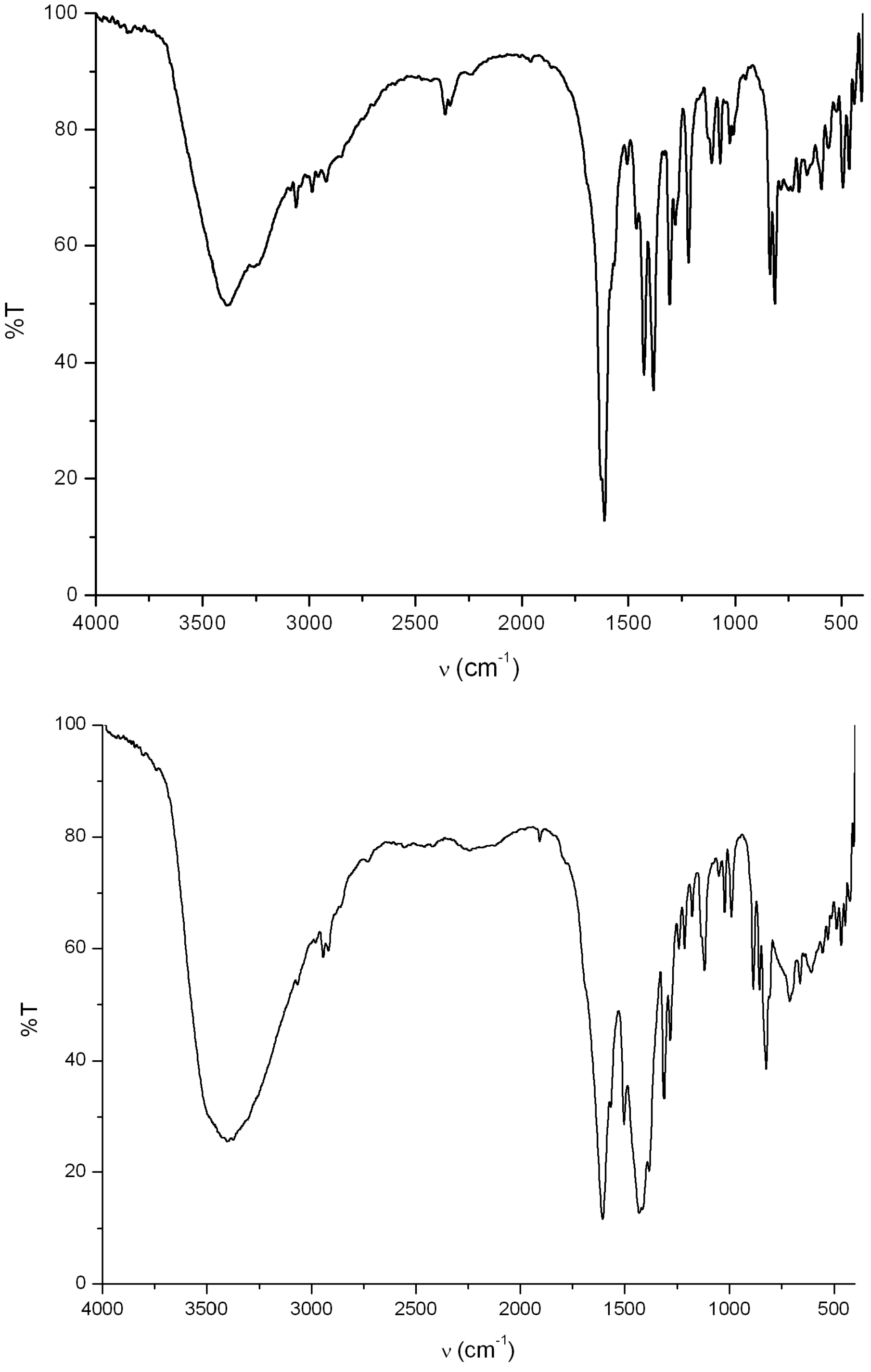

Figure S1. FT-IR spectra of compounds 1(top) and 2 (bottom).

Table S1. Experimental selected bond distances (Å) and angles (º) of the coordination environment of complexes **1** and **2**.

	**(1)**	**(2)**
Distances		
M – O(1)	2.070(3)	2.278(4)
M – O(2)	2.127(3)	2.321(5)
M – N(1)	2.147(3)	2.311(6)
Angles		
O(1) – M – O(2)	92.21(12)	85.72(16)
O(1) – M – N(1)	78.33(13)	72.83(17)
O(2) – M – N(1)	87.99(13)	92.10(19)

Table S2. Hydrogen-bonding geometry (Å, °)

**$ZnL_2(H_2O)_2$**				
Atoms	D – H	H···A	D···A	D - H···A
O(2)-H(2A) ···O(3)^{i}	0.85(5)	1.892(5)	2.736(6)	172.4(3)
O(2)-H(2B) ···H(6)^{ii}	0.85(5)	1.979(7)	2.821(5)	179.8(4)
**$CdL_2(H_2O)_2$**				
O(2)-H(2A) ···O(3)^{i}	1.073(6)	1.669(5)	2.716(7)	179.7(6)
O(2)-H(2B) ··H(6)^{ii}	1.091(5)	1.874(7)	2.684(5)	179.4(5)

Symmetry codes: (i) *x*; *y*; -1+*z*; (ii) 1-*x*, 1-*y*, 1-*z*

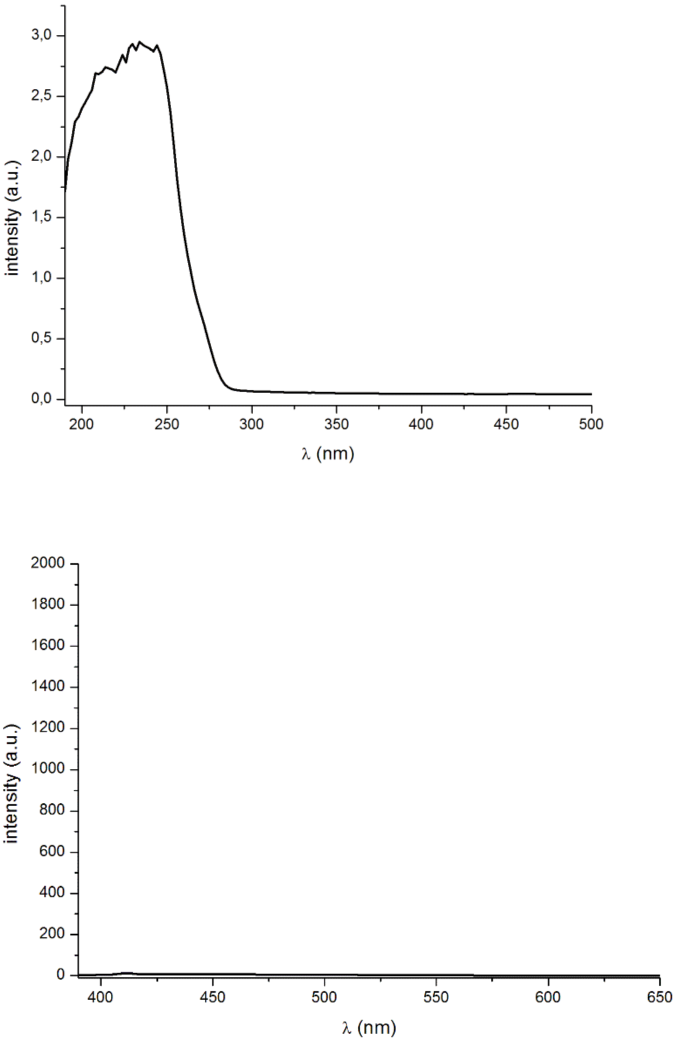

Figure S2. Electronic absorption (top) and emission (bottom) spectra of the ligand in diluted solution. Spectra were recorded in a 10^{-5}M of the ligand dissolved in distilled water.

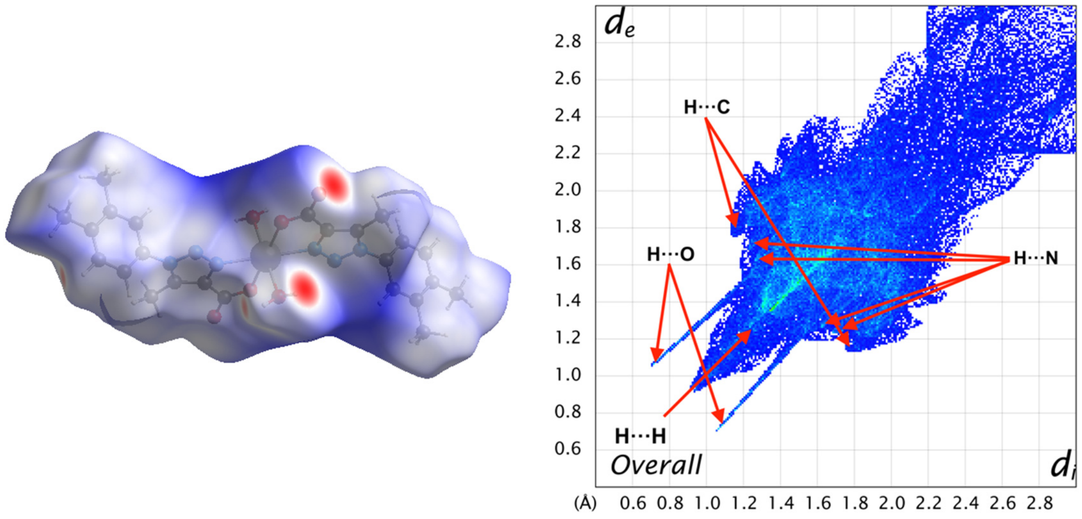

Figure S3. d_{norm} surface of $CdL_2(H_2O)_2$ complex (left) and its 2D fingerprint plot (right). In the left panel red and blue colors indicate strong and weak interactions, respectively. Isovalues ranges from -0.66 (blue) to +2.31 (red).

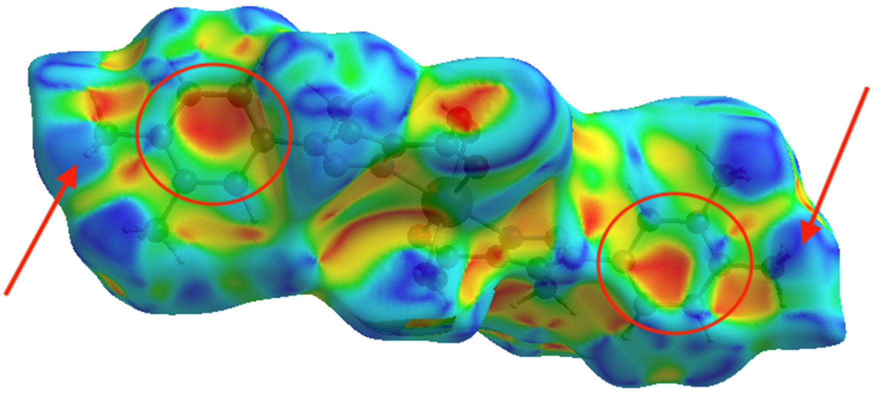

Figure S4. Shape index surface for $CdL_2(H_2O)_2$ complex. Red-orange and green-blue colors indicate weak and their recuprocal contacts, respectively. Isovalues ranges from -1.0 (red-orange) to +1.0 (green-blue).

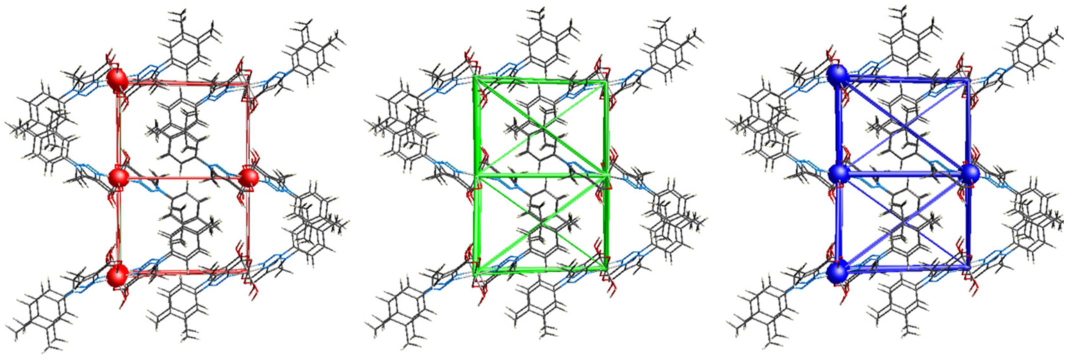

	E_{ele}	E_{dis}	E_{tot}
1	-208.2 kJ·mol^{-1}	-182.5 kJ·mol^{-1}	-313.4 kJ·mol^{-1}
2	-155.5 kJ·mol^{-1}	-174.2 kJ·mol^{-1}	-261.0 kJ·mol^{-1}

Figure S5. Energy framework diagrams for compound 1, and the respective energies for compounds 1 and 2.

The energy framework[1] was analysed to have a better understanding of the packing and topology of the crystal structure and the supramolecular rearrangement. According to the total contribution of intermolecular contacts, the supramolecular structure of these compounds is generated by an one-dimensional chain, directed by the translational symmetry elements in each compound along the *c*– axis, an alternated *zig-zag* chain along the *b*-axis, and layers along the *a*-axis, as consequence of two supramolecular structures described previously (see figure 6). This is due to the stabilization of these interactions driven by the M(II) ions, which act as a template reagent between the ligand units around them by the strong electrostatic interactions supported

by H···O interactions, where the M(II) ions use their special position in the crystals centro-symmetrical setting (0,0,0 and ½, ½, 0).

On the other hand, our calculations show that the interactions exhibit an approximately ladder shape energy topology. The low value of the electrostatic energy *versus* the dispersion energy in compound **1** can be attributed to the few classical hydrogen-bond interactions that generate the one-dimensional chain in the [001] direction, due to the strong contribution of H···O interactions that generates a SMOF structure. However, in the case of compound 2 the situation is different, because the values of E_{ele} and E_{dis} are nearly the same, which can be attributed to different VdW radii between Zn(II) and Cd(II). A similar behavior has been previously observed in crystal structures, even with changes in the crystal packing.[2]

(1) Mackenzie, C.F.; Spackman, P.R.; Jayatilaka, D.; & Spackman, M.A.; CrystalExplorer model energies and energy frameworks: extension to metal coordination compounds, organic salts, solvates and open-shell systems. *IUCrJ* **2017**, *4*, 575-587.

(2) Abtab, S. M. T., Audhya, A., Kundu, N., Samanta, S. K., Sardar, P. S., Butcher, R. J., ... & Chaudhury, M. (2013). Tetranuclear homo-(Zn^{II}_4 and Cd^{II}_4) and hetero-metal ($Zn^{II}_2Tb^{III}_2$ and $Cd^{II}_2Tb^{III}_2$) complexes with a pair of carboxylate ligands in a rare η^2:η^2:μ^4-bridging mode: syntheses, structures and emission properties. *Dalton Trans.* **2013**, 42, 1848-1861.

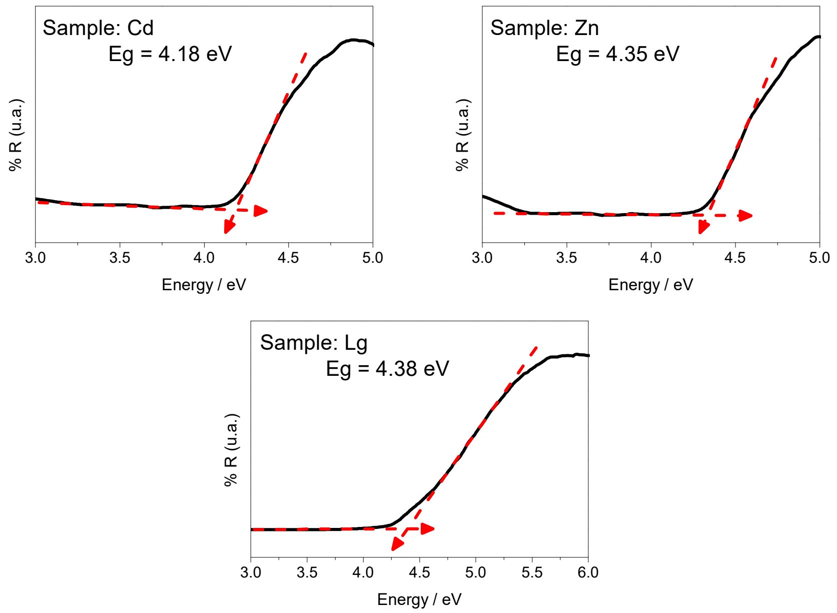

Figure S6. UV-Vis diffuse reflectance spectra of the ligand and the complexes. The values of the band gaps are also shown.

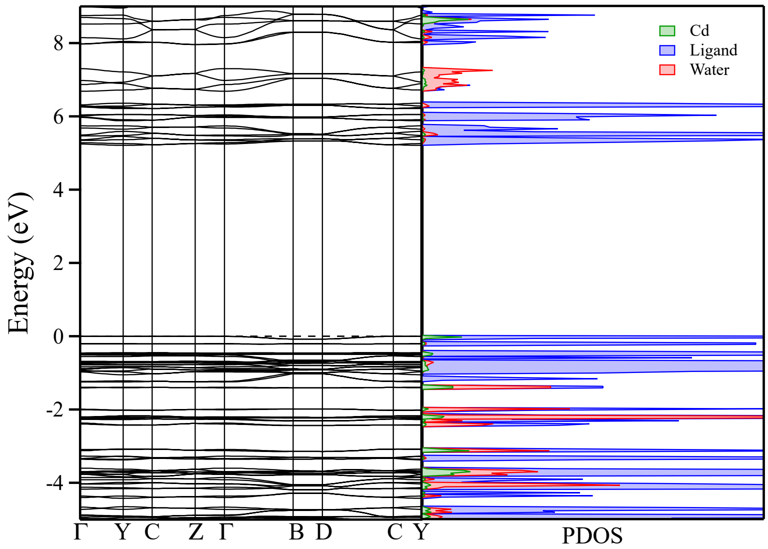

Figure S7. Band structure and partial density of states (PDOS) calculated with the B3LYP functional for compound **2**.

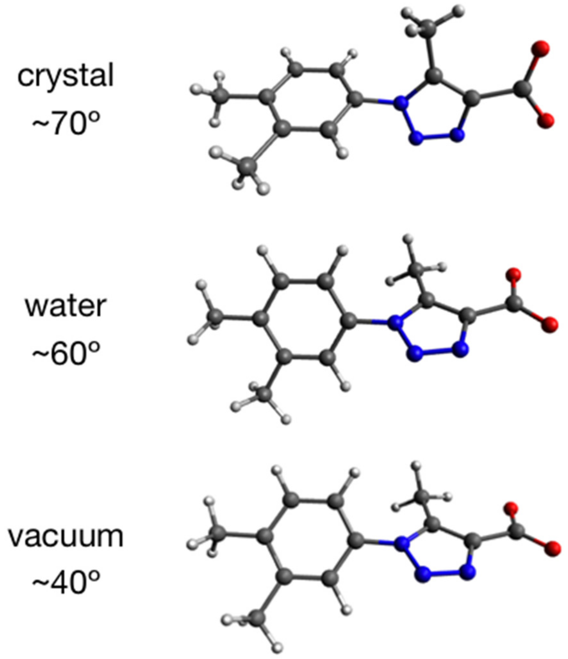

Figure S8. Optimized geometries for the isolated ligand anion L^- considering different environments. The labels indicate the magnitude of the dihedral angle between the phenyl and the triazole ring in each situation.

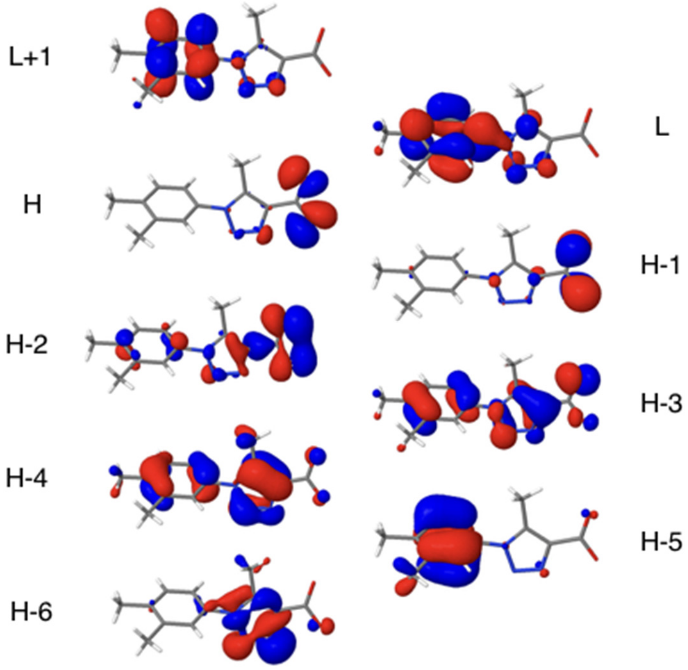

Figure S9. Frontier orbitals for the isolated ligand anion L^- in DCM (considering the experimental geometry obtained for the Zn CP).

main contributions	%	character
H-6 → L	7	n(N),π*
H-6 → L+1	2	
H-5 → L	3	π,π*
H-5 → L+1	4	
H-3 → L	20	π,π*
H-3 → L+1	4	
H-2 → L	15	n(O),π*
H-2 → L+1	2	
H-1 → L	8	n(O),π*
total	65	

Table S3. Main amplitudes and character for the S_5 state of the isolated ligand anion with the crystal structure of the Zn CP in DCM.

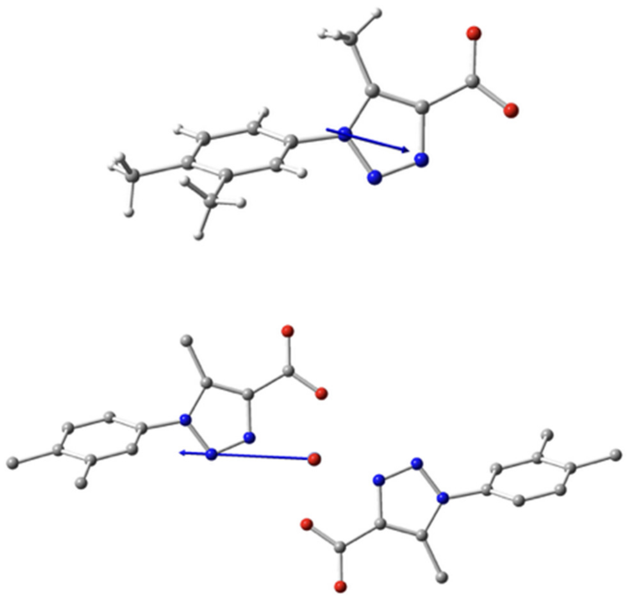

Figure S10. Transition dipole moment for the S_5 state of the ligand at the crystal geometry and for the S_5 of the Zn complex in the crystal geometry. In both cases a DCM environment has been considered. Origin, center of mass. The transition dipole moment has been scaled by a factor of 2.5.
